# Low Spigelian hernia in a 6-year-old boy presenting as an incarcerated inguinal hernia: a case report

**DOI:** 10.1186/1752-1947-3-34

**Published:** 2009-01-29

**Authors:** Efstratios Christianakis, Nikolaos Paschalidis, Georgios Filippou, Spiros Rizos, Dimitrios Smailis, Dimitrios Filippou

**Affiliations:** 1 Department of Pediatric Surgery, Pendeli Children's Hospital, Palaia Pendeli, Athens, Greece; 2 First Department of Surgery, Piraeus General Hospital "Tzaneio", Piraeus-Athens, Greece; 3 Department of Anatomy, Histology and Embryology, Nursing Faculty, University of Athens, Galatsi-Athens, Greece

## Abstract

**Introduction:**

Lower Spigelian hernia is a very rare entity. The clinical findings are similar to those of inguinal hernias and in many cases may be misdiagnosed. In the literature, only a few references to this entity have been reported in children. To the best of our knowledge, this is the first case report of a lower Spigelian hernia in a child who presented with an acute painful scrotum.

**Case presentation:**

We discuss the case of a 6-year-old Greek boy who presented to our emergency department complaining of severe pain in the left inguinal area and scrotum. The acute painful swelling started suddenly, without any obvious cause. The initial diagnosis was incarcerated inguinal hernia which was reduced with difficulty. Five days later, the patient still experienced mild pain during palpation and he was operated on. During the operation, a large lower Spigelian hernia was revealed and reconstructed.

**Conclusion:**

Although Spigelian hernias are rare in children and difficult to diagnose, physicians should be aware of them and include them in the differential diagnosis.

## Introduction

Lower Spigelian hernia is a rare entity in pediatric surgery, and is often misdiagnosed as an inguinal hernia [1]. Less than 50 cases have been presented in the literature, and only half of them occurred in younger children. In most of these cases, Spigelian hernias are considered congenital, although there is not enough evidence to support this suggestion [2,3]. We report a rare case of a lower Spigelian hernia in a child which presented as an incarcerated inguinal hernia.

## Case presentation

**Figure 1:**
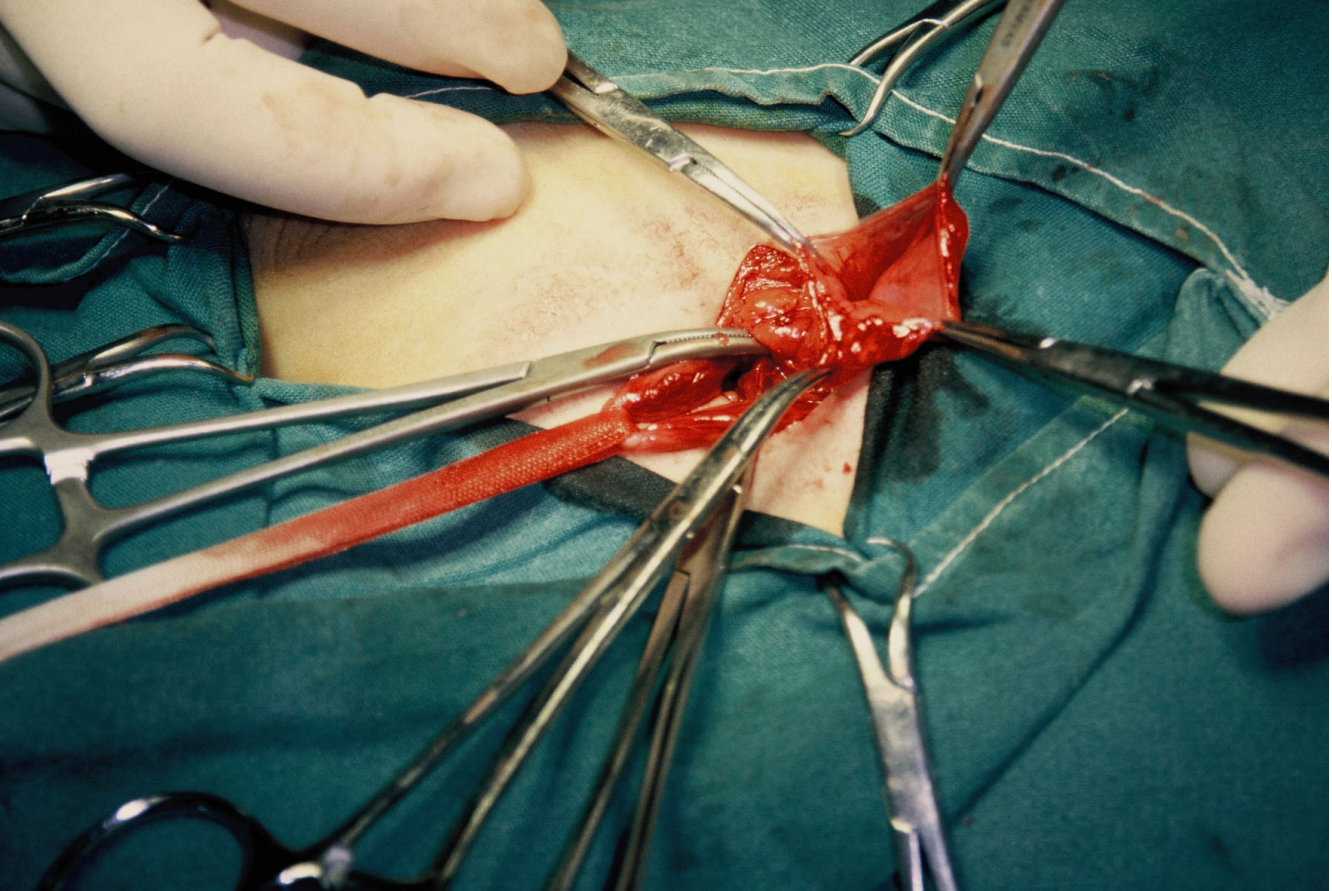
**Intra-operative photograph of the hernia showing the opened sac**.

A 6-year-old Greek boy presented with a painful intumescence of the left inguinal area. The child was initially referred to a general hospital where the physicians suggested that the problem was an incarcerated left inguinal hernia. The hernia was reduced with difficulty by the local surgeon and the next day, the child was referred to our hospital for further evaluation and treatment. In the physical examination, the left inguinal area appeared normal without signs of intumescence. The only findings were subcutaneous edema and a hematoma, probably due to the manipulations for the reduction. During palpation, the child presented with mild discomfort and pain in the left testicle without clinical evidence of testicular torsion. These symptoms persisted for 3 days and finally we decided to operate on the patient. A lower Spigelian hernia was identified during the operation. The hernia sac was found to penetrate from a small defect (with an estimated diameter of about 1.5 cm) in the Spigelian fascia within Hesselbach's triangle (Figure 1).

The lower Spigelian hernia orifice is usually located in a well-defined area. The defect develops as a ring-like opening through the fibers of the transversalis fascia and the fascia of the transversalis abdominus and internal oblique abdominus muscles. Spigelian hernias usually present just lateral to the rectus muscle in the lower left quadrant.

In our patient, the hernia sac was that of a typical Spigelian hernia. The orifice diameter was about 1.5 cm, while the sac extended up to 7 cm surrounded by preperitoneal fat (Figure 1). The hernia sac contained a small part of the large omentum which was reduced. Reconstruction of the hernia was performed with non-absorbable sutures. Eight years after the operation, the patient remains free of symptoms and recurrence.

## Discussion

Spigelian hernias occur through a congenital or usually acquired defect of the Spigelian fascia, lateral to the rectus muscle sheath [1]. In adults, almost 90% of acquired hernias occur within the anterior superior iliac spine and the umbilicus, while the majority of congenital hernias occur at the level of the arcuate line (fold of Douglas) [1-3]. Hernias that are protruding through the Spigelian fascia within Hesselbach's triangle, caudally and medially to the inferior epigastric vessels, are called low or lower Spigelian hernias [4]. Direct inguinal hernias can also occur in this area, although in most of these cases, the hernia's orifice is difficult to identify. This is the reason why many Spigelian hernias are misdiagnosed and usually considered as direct inguinal hernias, para-inguinal hernias or hernias through the conjoined tendon [5,6]. The diagnosis is usually made on the basis of the location of the hernia's orifice. The orifice of the Spigelian hernia is usually located inside the fibers of the transversalis fascia. The orifice is sharp and rigid in palpation and its diameter ranges from 2 to 3 cm [2,3,6]. It extends through this defect interstitially or penetrates the entire abdominal wall [7,8]. The sac of these hernias usually contains preperitoneal fatty tissue, and rarely omentum or intestine. Some authors have suggested that, in infants, Spigelian hernias may coexist with undescended testes.

The small size of the hernia neck may be a possible explanation for the increased incidence of incarceration. The incidence of incarceration varies significantly between various series and authors. Some authors have suggested that Spigelian hernia incarceration is a rare entity while others have suggested that it is very common with an estimated incidence of approximately 22% [9,10].

The pre-operative diagnosis of lower Spigelian hernias is difficult, especially in non-incarcerated cases. In most of the cases, diagnosis cannot be achieved by physical examination and the hernia can be easily misdiagnosed as congenital inguinal hernia. Recent studies have reported that the use of ultrasound and computed tomography (CT) may contribute to the pre-operative diagnosis in selected cases. A CT scan may be useful in the diagnosis of an incarcerated Spigelian hernia mainly due to its ability to demonstrate the layers of the anterior abdominal wall [11,12].

Reduction of the content with excision of the sac and closure of the fascia defect with non-absorbable sutures is the most common and widely accepted surgical approach, presenting the lowest recurrence rates [1,3,7]. Recent studies support the possible role of laparoscopy in the diagnosis and treatment of Spigelian hernia in children, suggesting that it may represent an acceptable therapeutic alternative [4,5].

## Conclusion

Spigelian hernia occurs through a congenital or, more often, an acquired defect in the Spigelian fascia, lateral to the rectus muscle sheath. It is very rare in children. The case that we present is unique because it is probably the first report of a lower Spigelian hernia presenting as acute scrotum. No similar cases have been reported in the literature. The aim of the present case report is to alert the physician of this rare entity because, although it is difficult to diagnose pre-operatively, accurate diagnosis and appropriate treatment are essential to avoid future recurrences.

## Abbreviations

SH: Spigelian hernia; CT: computed tomography.

## Competing interests

The authors declare that they have no competing interests.

## Authors' contributions

All authors contributed equally to the treatment of the patient (EC, NP, GF, DS, SR, DF). EC, NP and GF wrote the draft. SR and DF approved it. GF, DS and DF carried out the revision.

## Consent

Written informed consent was obtained from the patient's parents for publication of this case report and any accompanying images. A copy of the written consent is available for review by the Editor-in-Chief of this journal.
